# Investigating the relationship between specific executive functions and functional decline among community-dwelling older adults: results from a prospective pilot study

**DOI:** 10.1186/s12877-022-03559-6

**Published:** 2022-12-19

**Authors:** Emilie Verreckt, Elise Grimm, Stefan Agrigoroaei, Marie de Saint Hubert, Pierre Philippot, Gérald Cremer, Didier Schoevaerdts

**Affiliations:** 1 Geriatric Department, CHU UCL Namur, Site Godinne, Av. Dr. G. Therasse, 1, 5530 Yvoir, Belgium; 2 Psychological Sciences Research Institute, UCLouvain, Louvain-La-Neuve, Belgium

**Keywords:** Aging, Activities of daily living (ADL), Cognitive assessment, Autonomy, Inhibition, Flexibility, Planning, Updating in working memory

## Abstract

**Background:**

As cognitive functions and, more specifically, executive functions (EF) seem to influence autonomy among the elderly, we investigated the role of each of the five EF sub-components (inhibition, spontaneous flexibility, reactive flexibility, planning, and updating in working memory) for the risk of functional decline.

**Method:**

A total of 137 community-dwelling participants over 75 years of age were included in a prospective cohort study and assigned to three groups: individuals with neuro-degenerative cognitive disorders, those having cognitive disorders with non-degenerative aetiology, and a control group without any cognitive problems. We measured each EF sub-component and assessed functional decline by evaluating basic (b-ADL) and instrumental activities of daily living (i-ADL) at baseline and 6 months later. We conducted three separate multiple logistic regression models to examine the extent to which the five EF facets predicted overall functional decline at the end of the follow-up period.

**Results:**

We found that people who exhibited a decline in b-ADLs or/and i-ADLs over 6 months had worse performance on inhibition and two flexibility tasks than those who did not experience a decline. The results suggest that decliners have more difficulties in managing unforeseen events. Inhibition and updating in working memory predicted a decline in b-ADL while spontaneous and reactive flexibilities predicted a decline in i-ADL.

**Conclusion:**

In our sample, specific executive dysfunctions were associated with a decline in functional status. With respect to the risk of decline in b-ADL, deficits in inhibition may represent a risk factor, as it regulates over-learned activities. Bothtypes of flexibility, which allow the shifting and generating of adaptive responses, predicted decline in i-ADL. In sum, paying more attention to particular EF profiles would help clinicians to anticipate some aspects of functional decline.

**Supplementary Information:**

The online version contains supplementary material available at 10.1186/s12877-022-03559-6.

## Introduction

The number of patients who suffer from multiple medical problems is increasing as the average life expectancy rises. Consequently, earlier diagnosis and better prevention represent an important public health challenge. In recent years, links between co-morbidities, disabilities, and frailty have been extensively documented in the literature [1]. Functional decline has been defined as an active process linked to frailty [2], where physical, medical, psychological, social, and environmental factors are interwoven together [3]. This process explains the deterioration of self-care skills, where functional independence is reduced and disability is increased [4]. Functional status refers to potential functional limitations [5] which are restrictions in performing fundamental physical and mental actions [5]. These limitations are robust determinants of subsequent disability. As functional status has commonly been demonstrated in the literature to predict the length of hospital stays, hospital readmission rates, patient destination after a hospital stay and mortality, the evaluation of functional status has also become a public health challenge for prevention, early diagnosis, and management [3, 6]. Instrumental activities of daily living (i-ADLs) and basic activities of daily living (b-ADLs) are currently used to measure the level of autonomy and functional decline, as they represent basic physiological and self-maintenance needs [7–9].

As cognitive impairment is known to influence functional health, some authors have described a link between executive functions (EF) and functional decline, already in prodromal stages of Alzheimer’s disease [10]. More specifically, if EFs are now well known to decline with age, a large body of literature supports the association between EFs and functional decline in older adults [11–15]. Moreover, EF has been associated with other geriatric conditions, such as falls and frailty [16–18]. EFs are higher-ordered cognitive processes that orchestrate goal-planned behaviors when routines (self-regulation of automatic behaviors) are no longer sufficient. They play an important role in problem solving and in searching for adapted and adequate strategies to face new circumstances.

EF is associated with the prefrontal cortex and frontal-subcortical systems. They are known to decline with ageing and are potentially affected in a wide range of age-related diseases, such as Alzheimer’s disease (AD), Parkinson’s disease, cerebrovascular disease or depression [19–22].

Current neuropsychological models include 5 components of EF: inhibition, flexibility (divided in spontaneous flexibility and reactive flexibility), planning and working memory updating [23, 24]. These functions appear to be essential in managing activities of daily living and goal-directed behaviors. Inhibition involves controlling one’s attention, behavior, thoughts, and emotion to override a strong internal predisposition. This means controlling and preventing automatic and overlearned responses. In daily life, inhibition helps individuals exhibit self-control, resist temptation, resist impulsivity, and manage interference [25]. Spontaneous flexibility refers to the ability to generate ideas, solutions or memories. That function allows individuals to set up adequate responses to adaptive situations such as creating a new meal menu or changing clothes. As reactive flexibility refers to the ability to shift between different stimuli or response sets, we can consider its utility in daily activities that require switching from one task to another (also named switch attentional focus), such as facing changing circumstances. Planning refers to the ability to identify and organize the steps and elements needed to achieve a goal [26]. Finally, updating in working memory involves holding pieces of information in mind while refreshing them if necessary.

As mentioned before, there is strong evidence in the literature showing a significant correlation between EF and functional decline, suggesting that a lack of performance in one or more of these 5 EF components leads to impairments in some b-ADLs or i-ADLs [20, 27, 28]. Some authors, in an expert panel review from the American Neuropsychiatry Association, have suggested that functional capacities such as decision-making in medical or financial domains are strongly correlated with EF [28]. Moreover, some authors have shown an association between EF impairments, an increase in care needs and an increased risk of mortality among elderly women [29]. In a recent study, in a sample of AD patients, the results have revealed an association between performances in i-ADLs and specific EFs, such as the ability to inhibit a response, self-monitoring and set shifting (e.g., reactive flexibility) [14]. Yet, these authors did not find any significant relationship between i-ADLs and planning or between i-ADLs and problem-solving abilities, two important EF components. As the main limitation of these studies, the EF measure was either considered as a composite score or was simply reduced to a specific EF component, without a broader consideration of the other facets.

In contrast, in the present study we conceptualize EF as five distinguishable facets, in accordance with recent models [23, 24] and as considered in clinical practice. Indeed, in clinical practice, scientist-practitioners must identify which EF facet is impacted (by brain injury or brain illness) to understand daily life changes and to provide the most appropriate rehabilitation intervention. As EF facets interact with each other, considering them as separate dimensions represents one of the main contributions of our work. Even though this type of operationalisation and assessment is time consuming, we expect our approach to allow a better identification of specific neuropsychological mechanisms associated with loss of independence. Thus, the main purpose of the current study is to determine which of 5 executive functions, including inhibition, spontaneous and reactive flexibility, planning and updating in working memory, are involved in predicting functional decline 6 months later among adults over the age of 75. Additional knowledge about these cognitive processes underlying functional decline would help healthcare providers improve earlier detection, a more comprehensive geriatric assessment, and prevention of functional decline by adapted counselling and interventions.

## Methods

A longitudinal 6-month observational prospective cohort study was conducted at CHU UCL Namur, Godinne site, Belgium. Written informed consent was obtained before the first evaluation.

### Participants

Participants were recruited from the Centre Hospitalier Universitaire of UCLouvain (CHU UCL Namur ASBL, Godinne site), a tertiary university hospital located in the southern part of Belgium. They were all community-dwelling older adults, but some of them were hospitalized at the time of inclusion. All participants must have had more than 6 years of normal education. Individuals with hearing, visual, or language limitations that could constitute barriers to data collection were excluded. We also excluded individuals with Mini Mental Status Examination (MMSE) scores under 13/30 (to include those with important cognitive problems in our sample but who are still able to understand instructions).

### Measures

#### Assessment of functional decline

Functional status was assessed by the Belgian version of the Katz scale assessing 6 b-ADLs (each with 4 levels), with final scores ranging from 6 (complete independence) to 24 (complete dependence). The instrument is mostly based upon physical abilities and includes toileting, dressing and undressing, using toilets, eating, moving alone, and being continent [8]. The Lawton scale was used to assess i-ADLs, with final scores ranging from 9 (complete independence) to 27 (complete dependence) [7]. The i-ADL scale evaluates functional independence in nine domains (each with 3 levels) and includes using telephone, shopping, preparing meals, housekeeping, doing laundry, using car/public transportation, tinkering and maintaining one’s house, handling medication, and handling finances. Abilities assessed in i-ADL are currently known to involve high-level cognitive processes, while they also rely on physical capacities [30].

These scales were fulfilled at the time of inclusion by interview with a family member, caregiver or close contact (i.e., reliable informant). Six months after inclusion, functional status was re-assessed by phone with the same informant.

As the literature is heterogeneous in defining functional decline, we chose, like other authors, to consider functional decline as a worsening of one point in at least one domain evaluated by b-ADLs or i-ADLs between baseline and follow-up [3, 29, 31, 32].

#### Neuropsychological assessment

Neuropsychological assessment involved a global screening and a specific EF evaluation. All neuropsychological tests were administered by trained psychometricians during a 3-hour session.

The global cognitive screening was performed using the MMSE ranging from 0 to 30 [33]. Each of the five EFs was then evaluated by validated specific tests that are routinely used in clinical practice. We chose to record both time and number of errors in the 5 tasks to investigate quality and speed, both required in non-impaired EF. Inhibition was assessed using the Stroop Victoria Test [35], one of the numerous tasks based upon the interference effect described by Stroop [36], which, in the current version, has been adapted for elderly populations. In this task, the participant must name colored dots (red, blue, green, yellow) as quickly and accurately as possible. In a second condition, which represents low interference, dots are replaced by common words (when, hard, and, over) and the participant must name the color of each word. In the third condition, the high interference option, the color of each item has to be named, while the words now represent colors (red, blue, green, yellow) and do not correspond to the ink in which they were printed (for example: when the word “green” is printed in red, the subject must respond “red”). This task has been validated in the French language [34]. The independent variable was the number of errors in the strong interference subtest. Verbal fluency was employed to investigate spontaneous flexibility using scores (number of correct responses) on both lexical and semantic subtests [35, 36] in which subject has to provide as many words as possible beginning by a letter (“P”) or belonging to a category (i.e., animals).

Reactive flexibility was measured using the time to complete Trail Making Test-part B (TMT-B), as it is well known to assess shifting between multiple tasks or mental sets [23]. In this subtest, patients are presented an array of numbered and lettered circles and are asked to connect them in numerical and alphabetical order by alternating between both, as quickly as possible and without taking the pencil off the page. Planning was investigated by the Zoo map test, an ecological planning subtask from the Behavioral Assessment of Dysexecutive Syndrome and assessed by the profile score [37] calculated from realization time and correct responses. In the Zoo map test, participants are given the map of a zoo with a set of instructions relating the places they have to visit and rules they must stick to. Updating in working memory was assessed by the total score (number of correct responses) on a 2-back updating task (in which the patient must press a button whenever a pair of numbers-presented on the computer screen corresponds to the pair of numbers presented 2 trials before).

#### Covariates

Frailty was evaluated using the Frailty Fried Score (ranging from 0 indicating a non-frail status to 5 indicating a high level of frailty) [2]. The number of medications was also obtained during the interview. These two parameters were assessed, as they are currently considered risk factors for functional decline [17, 38]. Moreover, information about educational attainment was obtained and allowed the creation of two categories (secondary school or lower vs. higher than secondary school).

### Procedure

At baseline, after informed consent was obtained, demographic and medical data were recorded followed, on the same day, by neuropsychological testing. Informants completed b-ADL and i-ADL scales with clinicians on the same day.

Six months later, a new assessment was performed with the same informant by a phone call to measure functional status, b-ADL and i-ADL performance. They were completed even by participants who were admitted to a nursing home during the time of follow-up.

### Statistical analyses

To control for cognitive status in the analyses, we created a covariable that captures 3 groups of participants, based on the presence or absence of cognitive problems and the potential evolutive progression (neuro-degenerative aetiology, non-degenerative aetiology such as cerebrovascular disease or traumatic brain injury and a control group without any significant cognitive disorder).

To account for an alpha level of 0.05, an absolute precision estimate of ± 7%, a 95% confidence interval (CI), and an expected prevalence of functional decline of 35%, a sample size of 178 persons was initially determined. Categorical data from independent samples were compared using the Chi-square or Fisher’s exact tests, when appropriate. Numerical data from independent samples were compared with the independent-samples Mann-Whitney U test, as normality assumptions were not met. Participants with and without missing data were compared across all baseline variables using the independent-samples Mann-Whitney U test for continuous measures and Chi-square tests for categorical variables. All tests were two-tailed and performed with IBM Statistical Package for Social Sciences (SPSS software, version 25.0).

In univariate analyses, all *p*-values under 0.05 were considered statistically significant. The associations between all study variables were examined using bivariate Pearson correlations. Three separate multiple logistic regression models were then run to examine the extent to which the five executive functioning facets predicted overall functional decline 6 months later, specifically a decline in b-ADLs and i-ADLs. The models were controlled for age, sex, level of education, cognitive status according to three classifications (degenerative, non-degenerative or lack of cognitive disorder), number of medications, Frailty Fried score, and baseline b-ADL or i-ADL. Covariates were selected based on the significance of the Bravais-Pearson correlations or following evidence of an existing association documented in the literature. The Hosmer-Lemeshow test was used to evaluate the goodness-of-fit of the logistic regression models. We examined potential influences from existing outliers by saving the standardized residuals, examining Q-Q plots, and conducting sensitivity analyses with and without outliers (> 3 standard deviations (SDs)). With the results changing when outliers were removed, we report the models without the latter.

## Results

### Characteristics of included patients

Between 2014 and 2017, 179 individuals were included in the study. They were recruited through the geriatric day hospital or the ambulatory sector of the neurological department (*n* = 108; 60%), during their hospital stay (*n* = 20; 11%) or were external volunteers or accompanying patients (*n* = 51; 29%). Forty-two participants were removed from analyses due to missing data. Baseline demographics, clinical data and EF performance for the final sample (*n* = 137) are presented in Table 1. Participants were aged from 75 to 93 years old, and 54.7% of them were female. Using our main definition of functional decline (a worsening of 1 point in one of the two scales), 68.6% (95% CI: 60.1–76.3%) of the participants presented a functional decline (n = 94/137) over 6 months. At this 6-month follow-up, 35.8% (95% CI: 27.8–44.4%) and 58.4% (95% CI: 49.7–66.7%) of participants presented a functional decline in b-ADLs and/or i-ADLs, respectively (*n* = 49/137 and 80/137). At baseline and at the 6-month follow-up, participants presented mean b-ADL scores of 8.0 (*SD =* 3.2) and 8.5 (*SD =* 3.5) and mean i-ADL scores of 16.1 (*SD* = 5.1) and 18.3 (*SD* = 5.8), respectively.

**Table 1:** Comparison of the clinical profiles of participants experiencing (or not) a functional decline at 6 months of follow-up

Characteristics	Overall (*N* = 137)	Decliners (*N* = 94)	Non-decliners (*N* = 43)	*p*-value
Age years	81.00 (7.00)	82.00 (5.00)	80.00 (6.00)	***p *= .006**
Sex, N (% of Women)	75 (54.74)	50 (53.19)	25 (58.14)	*p* = .589
Education Level				*p* = .421
* Secondary school or lower*	77 (56.20)	55 (58.51)	22 (51.16)	
* Higher than secondary school*	60 (43.80)	39 (41.49)	21 (48.84)	
Cognitive Disorders				*p ***< .001**
* Absence of CD*	36 (26.28)	16 (17.02)	20 (46.51)	
* Non-neuro-degenerative CD*	33 (24.09)	20 (21.28)	13 (30.23)	
* Neuro-degenerative CD*	68 (49.63)	58 (61.70)	10 (23.26)	
Number of Medication,	7.00 (6.00)	7.50 (5.00)	6.00 (6.00)	*p ***= .046**
Frailty Fried score (total score/5)	2.00 (3.00)	2.00 (2.00)	1.00 (3.00)	*p ***= .009**
MMSE score (total score/30)	26.00 (5.00)	25.00 (6.00)	27.00 (5.00)	*p ***= .002**
Functional Status at baseline				
* b-ADL (total score/24)*,	7.00 (2.00)	7.00 (3.00)	6.00 (1.00)	*p ***= .001**
* i-ADL, (total score/27)*,	15.00 (8.00)	16.00 (8.00)	13.00 (8.00)	*p ***= .012**
Executive Function at Inclusion				
* Stroop (number of errors)*	4.00 (8.00)	5.00 (9.00)	2.00 (4.00)	*p ***< .001**
* Lexical fluency (total score)*	13.00 (9.00)	11.00 (8.00)	15.00 (12.00)	*p ***= .001**
* Semantic fluency (total score)*	20.00 (10.00)	17.00 (10.00)	23.00 (8.00)	*p ***< .001**
* TMT-B (time in seconds)*	249.00 (276.00)	263.00 (318.25)	178.00 (195.00)	*p ***= .001**
* Zoo profile (composite score)*	2.00 (2.00)	2.0 (2.00)	2.0 (1.00)	*p* = .071
* 2-Back (total Score/16)*	10.00 (6.00)	9.50 (6.00)	11.00 (6.00)	*p* = .108

Numerical data are expressed as the median and interquartile range. Categorical data are expressed by their absolute number and their percentage. Significant independent Whitney-Mann U Test or Chi-squared tests in bold

*M* median, *CD *cognitive disorders, *MMSE *Mini Mental State Examination, *TMT-B *Trail Making Task B, *b-ADL *basic activities of daily living, *i-ADL* instrumental activities of daily living

Table 1 presents the characteristics of individuals who declined at the end of follow-up, those who did not experience a functional decline and the entire cohort. According to univariate analyses, compared to participants who did not experience functional decline on either scale, participants who did were more likely to have more errors on the Stroop test, lower lexical and semantic fluency scores, and longer TMT response times. They presented a higher b-ADL and i-ADL score at baseline, were also older, were more frequently presenting a cognitive disorder or a lower MMSE score, were taking more medication and presented a higher level of frailty (Frailty Fried score). Individuals who declined only on the b-ADL scale had lower lexical fluency scores and made more errors on the Stroop task. Individuals who declined on the i-ADL scale more frequently presented a neuro-degenerative disease, a lower MMSE score, a higher level of frailty and presented more inhibition errors, a lower spontaneous flexibility performance and response time in reactive flexibility (see Supplementary Table 1).

### Predictors of functional decline

Table 2 shows the inter-correlations among all study variables. The correlation between b-ADLs or i-ADLs at baseline and the results of EF tasks are mostly and globally moderate. Significant correlations could also be observed among all five executive functioning facets. Only the results of spontaneous flexibility tests were not associated with planning. In the multivariate analyses results shown in Table 3, predictors of functional decline in *b*-ADLs included a higher number of errors on the Stroop task (*p =* .046) and a higher 2-back score (*p* = .007) after adjustment for age, sex, education level, the presence of cognitive disorders, number of medications, Frailty Fried score and baseline b-ADLs Similarly, lower baseline i-ADL (*p* < .001), lower semantic fluency scores (*p* = .015) and longer times on the TMT-B task (*p =* .007) significantly predicted functional decline in i-ADL 6 months later. With respect to overall functional decline (i.e., decline in one of the scales), lower semantic fluency scores (*p* = .048) and the presence of a neuro-degenerative disorder (*p* = .014) emerged as significant predictors

**Table 2:** Intercorrelations among all study variables included in the logistic regression models

	1	2	3	4	5	6	Baseline b-ADL	6-months b-ADL	Baseline i-ADL	6-months i-ADL	11	12	13	14	15
1. Age	1														
2. Sex	0.00	1													
3. Education	− 0.14	0.14	1												
4. Group	− 0.17*	− 0.03	0.14	1											
5. Medication	0.17*	− 0.09	− 0.14	− 0.18*	1										
6. Fried score	0.14	− 0.02	− 0.20*	− 0.13	0.38***	1									
7. Baseline b-ADL	0.23**	− 0.06	− 0.11	− 0.06	0.34***	0.48***	1								
8. 6-month b-ADL	0.25**	0.01	− 0.06	− 0.13	0.29**	0.41***	0.74***	1							
9. Baseline i-ADL	0.29**	− 0.01	− 0.15	− 0.29**	0.19*	0.31***	0.46***	**0**.47***	1						
10. 6-month i-ADL	.39***	0.10	− 0.16	− 0.29**	0.24**	0.37****	0.46***	0.55***	0.72***	1					
11. Stroop Errors	0.17	0.01	− 0.14	− 0.18*	0.26**	0.39***	0.31***	0.40***	0.35***	0.52***	1				
12. Lexical fluency	− 0.28**	0.01	0.26**	0.31***	− 0.27**	− 0.32***	− 0.28**	− 0.29**	− 0.34***	− 0.47***	− 0.44***	1			
13. Semantic fluency	− 0.35***	− 0.05	0.23**	0.23**	− 0.06	− 0.23**	− 0.29**	− 0.30***	− 0.43***	− 0.55***	− 0.35***	0.45***	1		
14. TMT-B time	0.17*	0.04	− 0.11	− 0.18*	0.19*	0.16	0.24**	0.15	0.41***	0.43***	0.37***	− 0.35***	− 0.35***	1	
15. Zoo profile	− 0.11	0.11	0.17*	0.21*	− 0.07	− 0.10	− 0.25**	− 0.25**	− 0.29**	− 0.27**	− 0.32***	0.16	0.16	− 0.21*	1
16. 2-Back score	− 0.20*	0.04	0.20*	0.16	− 0.15	− 0.21*	− 0.42***	− 0.23**	− 0.44***	− 0.41***	− 0.41***	0.35***	0.39***	− 0.41***	0.34***

*p < .05; ** *p* < .010; ***p < .001; sex (0 = women; 1 = men); education (0 = secondary school or lower; 1 = higher than secondary school); group (1 = non-degenerative disorder; 2 = degenerative disorder; 3 = no disorder); ADL = activities of daily living; TMT = Trail-Making Test. The values in bold indicate significant coefficients

**Table 3:** Predictors of functional decline in b-ADL, i-ADL or either one after 6 months of follow-up, using multiple logistic regression analyses

	b-ADL Model				i-ADL Model				Overall Model (b-ADL and i-ADL)					
Baseline Measures	Odds ratio	95% Confidence Intervals		*p*-value	Odds ratio	95% Confidence Intervals		*p*-value	Odds ratio	95% Confidence Intervals		*p*-value		
Age (years)	1.02	0.92	1.13	0.703	1.04	0.93	1.16	0.465	0.97	0.86	1.10		0.674	
Sex	0.84	0.38	1.85	0.668	0.63	0.26	1.54	0.313	0.75	0.28	2.00		0.571	
Education	0.74	0.32	1.69	0.469	0.87	0.34	2.26	0.776	0.72	0.25	2.09		0.548	
Cognitive disorders														
*Non-degenerative*	1.32	0.39	4.51	0.657	1.11	0.31	3.98	0.873	0.82	0.21	3.11		0.764	
*Degenerative*	2.32	0.72	7.43	0.157	2.96	0.85	10.36	0.089	**5.67**	**1.43**	**22.49**		**0.014**	
Medication (number)	1.04	0.91	1.18	0.583	1.12	0.97	1.30	0.119	1.09	0.93	1.26		0.299	
Frailty Fried score	1.19	0.85	1.66	0.323	1.40	0.96	2.06	0.084	1.34	0.87	2.05		0.187	
b-ADL	0.99	0.85	1.16	0.885	-	-	-	-	1.16	0.90	1.50		0.239	
i-ADL	-	-	-	-	**0.77**	**0.66**	**0.89**	**< 0.001**	0.92	0.80	1.06		0.258	
Stroop errors	**1.09**	**1.00**	**1.19**	**0.046**	1.08	0.96	1.21	0.197	1.14	0.98	1.32		0.095	
Lexical fluency	0.98	0.91	1.05	0.566	0.99	0.92	1.07	0.796	0.98	0.90	1.05		0.528	
Semantic fluency	0.98	0.91	1.05	0.512	**0.90**	**0.83**	**0.98**	**0.015**	**0.92**	**0.85**	**1.00**		**0.048**	
TMT-B time	1.00	1.00	1.00	0.900	**1.00**	**1.00**	**1.01**	**0.007**	1.00	1.00	1.01		0.310	
Zoo profile	0.92	0.65	1.30	0.626	0.79	0.53	1.17	0.233	0.82	0.53	1.27		0.369	
2-Back score	**1.18**	**1.05**	**1.34**	**0.007**	1.05	0.92	1.19	0.508	1.17	1.00	1.37		0.054	

Significant p-values in bold

*ADL* Activities of daily living, *TMT* Trail-Making Test

B-ADL model: *R*^2^ = 0.21, Hosmer-Lemeshow test, *p* = .063. I-ADL model: *R*^2^ = 0.44, Hosmer-Lemeshow test, p = .176. Overall model: *R*^2^ = 0.45, Hosmer-Lemeshow test *p* = .93

### Assessment of subjects with missing data

They were significant differences between individuals who were excluded from analyses (*n* = 42) and those who were included (*n* = 137); namely, those with missing data were more likely not to have cognitive disorders [*X*^2^(2, 179) = 51.26, *p* < .001] and were more likely to have lower Frailty Fried scores (median = 0.00 vs. 2.00, *U* = 4108.00, z = 4.63, *p* < .001), higher MMSE scores [median = 28.00 vs. 26.00, *U* = 1289.50, *z*=-5.43, *p* < .001], higher baseline b-ADL [median = 8.00 vs. 7.00, *U* = 1680.00, *z*=-4.22, *p <* .001] and lower i-ADL [median = 9.00 vs. 15.00, *U* = 4507.50, *z =* 5.58, *p* < .001]. With respect to cognitive variables, individuals with missing data were more likely to make less errors on the Stroop task [median = 0.00 vs. 4.00, *U* = 4497.00, *z* = 5.56, *p* < .001], to achieve better scores on lexical [median = 17.50 vs. 13.00, *U* = 1796.50, *z*=-3.68, *p* < .001] and semantic [median = 22.00 vs. 20.00, *U* = 2056.50, *z*=-2.80, *p* = .005] fluency tests, to respond faster to the TMT-B [median = 154.50 vs. 249.00, *U* = 3893.50, *z* = 4.67, *p* < .001], and to have both better Zoo profiles [median = 3.00 vs. 2.00, *U* = 1744.50, *z*= -4.00, *p* < .001)] and better 2-back sores [median = 13.00 vs. 10.00, *U* = 1493.00, *z*=-4.73, *p* < .001] compared to individuals without missing data.

## Discussion

This study investigated whether specific EF sub-components predict functional decline over 6 months in a community-dwelling population aged over 75 years. Considering EF as 5 interactive sub-components and not as a single entity allows a new approach to better understand the links between functional decline and specific cognitive processes. Thus, our approach generated additional knowledge, in contrast with previous studies linking EF as a global component and functional decline [39]. By considering the 5 distinct cognitive facets of EF (i.e., inhibition, spontaneous flexibility, reactive flexibility, planning, and updating in working memory) we increase the understanding of the links between EF and functional health. To the best of our knowledge, only three previous studies have examined specific EF dimensions (reactive flexibility, updating in working memory, and planning) and functional decline [29, 40, 41]. However, none of these previous studies has examined the full array of EF sub-components.

Our results show a moderate relationship between each EF and both basic and instrumental facets of ADL at baseline and at 6 months of follow-up. Similar findings were found by other authors linking functional status and some EF (or EF as a whole) in normal and pathological ageing [12, 13, 15, 42]. In the same direction, we also found a significant correlation between b-ADLs and i-ADLs and age, medication, education level and frailty.

Even if we consider that EF facets interact in real life and the issue of EF tasks impurity [23], our results indicate that specific sub-components of EF are linked to functional status measured by both ADL scales. Moreover, when subdividing subjects into 2 groups (the ones who decline across 6 months -decliners- and the ones who don’t -non decliners-), we showed that decliners present less performance on inhibition and both spontaneous and reactive flexibilities. These results suggest that decliners will present more difficulties when confronted with unforeseen events/situations. Considering functional decline as a change in daily living, we can easily understand that decliners will be confronted with a double problem: while dependency tends to increase, these individuals have fewer adaptation abilities. If we want to see upstream of these implications, and according to multivariate analysis, some EF processes appear to predict functional decline independently. Our results indicate that inhibition and updating in working memory predict a decline in b-ADLs, while reactive or spontaneous flexibility are more associated with a decline in i-ADLs.

In our study, increasing inhibition errors may be considered a risk factor for functional decline in b-ADLs. Jefferson considered inhibition (time in the Stroop interference assessment is regarded as an independent variable) as a risk factor for functional impairment in healthy older adults without considering functional decline [43]. Additionally, in a 1-year follow-up study, Boyle showed that EF studied globally (including inhibition, both flexibilities and motor programming) without differentiating executive processes also predicted functional decline in a group of patients suffering from vascular dementia [44]. Another issue is the link between inhibition and gait patterns. Hausdorff has shown that gait behaves like a complex motor task (such as catching a moving object) [45]. More specifically, they showed that gait (stride time variability) was strongly related to an object catching task (which is known to require a high executive component) but also to a composite score of the Stroop test. As this latter is known to evaluate cognitive inhibition, we understand that this specific cognitive function will play a role even in basic motor management.

Our finding that increasing qualitative abilities in updating working memory predict functional decline in b-ADLs was surprising. We did not find any other studies linking updating in working memory and functional decline in healthy adults, even if updating was shown to be a significant mediator between age and fluid intelligence [46]. In contrast, we should have expected that increasing working memory ability would play a protective role in activities of daily living, as was shown among patients suffering from AD, in whom high working memory performance (digital span) was associated with a slower rate of functional decline [40]. However, it is important to note that the correlation coefficients (both Bravais-Pearson and Spearman) between updating and all four indexes of functional status (at baseline and at 6 months) were all negative and significant. On its own, higher updating ability was associated with an increased functional status, as originally predicted. That’s in the presence of the other predictors that a positive association between updating and functional decline emerged. Hence, we believe that further work is required to understand the role of updating in interaction with the covariates included in our regression models. In contrast, for the other EF facets, the directionality of the associations with functional decline remains the same in the correlational and regression models.

Furthermore, other data have stressed that impairment in working memory represents a risk factor for poor medication adherence among older adults living at home [47]. Updating in working memory enables us to maintain and refresh a data flow in short-term memory. If the influence of updating on working memory on i-ADLs (which are sustained by high-level cognitive processes) is more predictable, its interaction with b-ADLs is less clear because b-ADLs require more automatic and over-learned routines. In the future, it might be interesting to investigate the hypothesis that people with greater working memory ability have a better awareness, evidenced by confrontation and treatment in working memory regarding daily difficulties; consequently, these individuals will call for help earlier.

We found a link between reactive flexibility and baseline functional status or a decline in i-ADLs. Time in reactive flexibility, as the capacity to shift from one stimulation to another, may be considered a risk factor for functional decline in i-ADLs. According to our results, Johnson et al. also showed an association of EF dysfunction measured by TMT-B and changes in b-ADL and i-ADL among women over 65 years [29]. The limitation of that study was the lack of inclusion of other EF sub-components. Four years later, also looking at reactive flexibility as a single domain, Marshall confirmed results showing that executive dysfunction is a key factor in i-ADL impairment [48].

Decreased performance in generating semantic information, supported by spontaneous flexibility, also represents a risk factor for functional decline in i-ADLs. In the literature, awareness of cognitive impairment has been linked with verbal fluency, which is associated with language and spontaneous flexibility, especially among AD patients [49]. Regarding spontaneous flexibility, Martyr linked impaired verbal fluency to a more dependent auto-evaluation in i-ADLs [50]. The authors argued that this language feature in cases of early dementia explains their heightened awareness of their disabilities (troubled consciousness) and consequently led to better compliance. In clinical terms, this supposes that reduced flexibility performance either in the ability to shift attention from one stimulus set to another or/and in generating semantic information, as is usually tested in our conventional executive tests, must alert clinicians of a potential risk of a decrease in the ability to manage instrumental activities of daily living 6 months later.

In clinical practice, if flexibility has an impact on functional status, either by the difficulty of switching from one task to another (for example, stopping an administrative task to answer the phone) or by the difficulty in generating concepts that are useful for tasks in progress (for example, listing ingredients needed for a recipe), we must also expect that one or both of these difficulties are announcing, 6 months later, a need to support the person in some activities of daily living, such as those listed in the Lawton questionnaire, i.e., using the telephone, shopping, preparing meals, housekeeping, laundry and dressing, using car/public transportation, handling medication and managing finances.

We raise the question of why flexibility predicts a decline in i-ADLs specifically. It is known that i-ADLs require high-level processes and that, as a sub-component of EF, both flexibilities are part of these high-level processes [14, 51, 52]. More specifically, when problem solving is needed or when facing unforeseen events, the difficulty of generating adequate scripts/solutions or the difficulty of changing the operating mode will impair instrumental tasks (more than would inhibition of an automatic response or the ability to manipulate in working memory), thus leading to apathy signs or perseverance in inadequate behavior. B-ADLs are described as more motor and automatic tasks than i-ADLs, requiring less high-level cognitive processes [13]. These activities will therefore preferentially decrease when failing to prevent the appearance of over-learned routines (to replace by more suitable scripts) when situations require adaptive behaviors. When adaptive behavior is needed or when faced with unforeseen events, inhibiting over-learned scripts will then allow the flexibility processes to generate another script (i.e., spontaneous flexibility) more suitable and/or shift from a treatment mode to another (i.e., reactive flexibility). The first part of this situation, related to inhibition, will explain the decline in b-ADLs. The second part, requiring flexibility, will explain a decline in i-ADLs. Updating in working memory will maintain data and allow fluid flow, which is required for adapted behavioral scripts. The best management of this flow of information and the propensity of not being able to prevent automatisms may explain a decrease in b-ADLs, which is more related to our own body. We may hypothesize that these over-learned activities are stronger and therefore more automated. Consequently, when the context requires adaptation, resources in inhibition have to be greater to prevent their appearance in b-ADLs compared to i-ADLs (as i-ADLs require fewer overlearned scripts and are more confronted with adaptive and unforeseen situations). Inhibition as a risk factor can be interpreted according to the strength of automatic patterns related to our own body that must be inhibited (stronger in b-ADLs than in i-ADLs).

Finally, while planning has already been mentioned in the literature as a predictor of functional decline in older adults [41], the results of our study did not reveal a significant association. Possible explanations could be related to the nature of the task (Victoria Stroop Test vs. original Stroop task, for example) and the characteristics of the samples (participants with higher socioeconomic status were used in previous work).

The current study presents several limitations. First, we excluded 23% of eligible persons due to missing data, which may challenge the representativeness of the sample. A reduction of our sample size may have led to a type II error, meaning wrongly failing to reject the null hypothesis. Many of our dropouts were due to incomplete data (performances on functional or neuropsychological scales, medication data). To question the sample representativeness, we compared subjects with missing data to the final sample: excluded patients were more likely to not present any cognitive disorder (showed higher MMSE scores), to be more fit (lower Fried and i-ADL scores) and to present better executive functioning performances. This may represent a selection bias, as dropouts may represent healthier people, who may show different processes in functional decline. Furthermore, coupled with a limited sample size, these elements lead us to consider our work as a pilot study.

Second, for the sake of ecological validity, in our study, we focused on a heterogeneous sample of patients (any cognitive trouble, non-evolutive cognitive disorders, neuro-degenerative illness) and did not differentiate them according to physio pathogenesis. In future investigations, it might be useful to consider these 3 groups independently. Also, our sample included patients who were either hospitalized or not at baseline. As hospitalization represents a risk for functional decline, this variable should be systematically considered in future studies.

Third, we chose a 6-month follow-up period, which might be a rather short time. Even if significant results were observed, a longer period of follow-up would be important to confirm predictors of functional decline in the long term.

Future investigations might better define the impact of each specific EF according to each specific task included in either b-ADLs or i-ADLs and their relationship with a later functional decline. We suggest to evaluate EF at follow-up to bring complementary data to our analyses, more prospective investigations are also necessary to corroborate our hypothesis.

## Conclusion

In a sample of community-dwelling old adults aged over 75 years, EF was linked with a later functional decline in b-ADLs or i-ADLs after 6 months of follow-up. More specifically, inhibition, updating in working memory, spontaneous or reactive flexibilities were important predictors of functional decline, even after adjustment with some covariates. Even if the existing literature had conclusively established a clear link among EF, analyzed globally, and functional decline, our study adds another interesting approach to assess the role of different specific EFs in the loss of autonomy. We hypothesize that inhibition will influence the course of b-ADLs by managing the performance of over-learned activities, while flexibility will explain a decrease in i-ADLs by generating and shifting to adaptive responses. Updating in working memory would, according to our interpretation, explain individuals’ better awareness and earlier calls for help leading to the enforcement of better adaptive solutions and prevention in order to continue living at home and that can be helpful during counselling sessions. As implementing good health behaviors and an individualized plan of care represent a real challenge in older populations, and as EFs are needed for adaptive behaviors, it appears evident to integrate these cognitive risk factors for functional decline in our daily clinical practice and comprehensive geriatric assessments.

## Supplementary Information

**Additional file 1: Supplementary Table 1.** Descriptions of the clinical profiles of participants experiencing functional decline at 6 months of follow-up on either b-ADL or i-ADL.

## Data Availability

The dataset used for the analyses in the current article are available on OSF (https://osf.io/6ux8y/).
