# Supplementary material for: Investigating the relationship between specific executive functions and functional decline among community-dwelling older adults: results from a prospective pilot study
Source: BMC Geriatr. 2022 Dec 19;22:976. doi: 10.1186/s12877-022-03559-6 (PMC9762049; doi:10.1186/s12877-022-03559-6)
Supplement: Supplementary file 1 — Additional file 1: Supplementary Table 1. Descriptions of the clinical profiles of participants experiencing functional decline at 6 months of follow-up on either b-ADL or i-ADL. [file 12877_2022_3559_MOESM1_ESM.docx]

**Supplementary Table 1. Descriptions of the clinical profiles of participants experiencing functional decline at 6 months of follow-up on either b-ADL or i-ADL**

| Characteristics | Decliners on b-ADL (N=49) | Non-decliners on b-ADL (N=88) | Statistical test | Decliners on i-ADL (N=80) | Non-decliners on i-ADL (N=57) | Statistical test |
| --- | --- | --- | --- | --- | --- | --- |
| Age years | 82.00 (6.00) | 81.00 (6.00) | U=2438.00, z=1.27, p=.204 | 82.50 (6.00) | 80.00 (6.00) | U=2902.50, z=2.73, **p=.006** |
| Sex, N (% of Women) | 25 (51.02) | 50 (56.82) | X^2^(1,137) = 0.43, p=.513 | 41 (51.25) | 34 (59.65) | X^2^(1,137) = 0.95, p=.330 |
| Education level |  |  | X^2^(1,137) = 0.04, p=.846 |  |  | X^2^(1,137) = 1.13, p=.289 |
| Secondary school or lower | 27 (55.10) | 50 (56.82) |  | 48 (60.00) | 29 (50.88) |  |
| Higher than secondary school | 22 (44.90) | 38 (43.18) |  | 32 (40.00) | 28 (49.12) |  |
| Cognitive disorders |  |  | X^2^(2,137) = 5.02, p=.081 |  |  | X^2^(2,137) = 10.00, **p=.007** |
| Absence of CD | 8 (16.33) | 28 (31.82) |  | 14 (17.50) | 22 (38.60) |  |
| Non-neuro-degenerative CD | 11 (22.45) | 22 (25.00) |  | 18 (22.50) | 15 (26.31) |  |
| Neuro-degenerative CD | 30 (61.22) | 38 (43.18) |  | 48 (60.00) | 20 (35.09) |  |
| Number of medications, | 7.00 (6.00) | 7.00 (6.00) | U=2446.00, z=1.31, p=.191 | 8.00 (6.00) | 6.00 (6.00) | U=2715.50, z=1.91, p=.056 |
| Frailty Fried score (total score/5) | 2.00 (2.00) | 1.00 (3.00) | U=2561.50, z=1.86, p=.062 | 2.00 (2.00) | 1.00 (3.00) | U=2865.00, z=2.62, **p=.009** |
| MMSE score (total score/30) | 26.00 (6.00) | 25.50 (6.00) | U=1913.50, z=-1.09**,** p=.274 | 24.00 (6.00) | 26.00 (5.00) | U=1502.00, z=-3.41**, p=.001** |
| Functional status at baseline |  |  |  |  |  |  |
| b-ADL (total score/24), | 7.00 (4.00) | 6.50 (2.00) | U=2420.50, z=1.26**,** p=.209 | 7.00 (4.00) | 6.00 (1.00) | U=2925.50, z=2.98**, p=.003** |
| i-ADL, (total score/27), | 18.00 (9.00) | 14.50 (7.00) | U=2679.50, z=2.36, **p=.018** | 15.50 (8.00) | 15.00 (9.00) | U=2418.00, z=0.60, p=.546 |
| Executive function at inclusion |  |  |  |  |  |  |
| Stroop (number of errors) | 6.00 (10.00) | 3.00 (7.00) | U=2906.50, z=3.38, **p=.001** | 5.00 (9.00) | 2.00 (6.00) | U=2973.50, z=3.04, **p=.002** |
| Lexical fluency (total score) | 11.00 (6.00) | 14.00 (12.00) | U=1713.50, z=-1.99, **p=.047** | 10.50 (9.00) | 15.00 (11.00) | U=1623.00, z=-2.87, **p=.004** |
| Semantic fluency (total score) | 18.00 (11.00) | 21.00 (9.00) | U=1727.00, z=-1.93, p=.054 | 17.00 (10.00) | 22.00 (10.00) | U=1505.50, z=-3.39, **p=.001** |
| TMT-B (time in seconds) | 256.00 (304.00) | 246.00 (255.75) | U=2486.50, z=1.48, p=.138 | 283.00 (329.75) | 193.00 (177.00) | U=3134.50, z=3.73, **p<.001** |
| Zoo profile (composite score) | 2.00 (2.00) | 2.00 (2.00) | U=1979.00, z=-0.83, p=.409 | 2.00 (2.00) | 2.00 (1.00) | U=2000.50, z=-1.27, p=.205 |
| 2-Back (total score/16) | 10.00 (5.00) | 10.50 (6.00) | U=2346.50, z=0.86, p=.391 | 9.00 (7.00) | 11.00 (5.00) | U=1909.50, z=-1.62, p=.105 |

*Notes.* Numerical data are expressed as the median and interquartile range. Categorical data are expressed by their absolute number and their percentage. Significant independent Whitney-Mann U Test or Chi-squared tests in bold. M=median; CD=cognitive disorders; MMSE=Mini Mental State Examination; TMT-B=Trail Making Task B; b-ADL=basic activities of daily living; i-ADL=instrumental activities of daily living.
